# Investigation of the Impacts of Thermal Shock on Carbon Composite Materials

**DOI:** 10.3390/ma12030435

**Published:** 2019-01-31

**Authors:** Wenfu Wei, Yijun Song, Zefeng Yang, Guoqiang Gao, Pan Xu, Ming Lu, Chuanjun Tu, Mingli Chen, Guangning Wu

**Affiliations:** 1 School of Electrical Engineering, Southwest Jiaotong University, Chengdu 610000, China; wfwei@swjtu.edu.cn (W.W.); syj_swjtu@163.com (Y.S.); xnjdggq@home.swjtu.edu.cn (G.G.); swjtuxp@163.com (P.X.); 18602830029@163.com (M.L.); gnwu@swjtu.cn (G.W.); 2 School of Material, Hunan University, Changsha 410000, China; cqc@my.swjtu.edu.cn; 3 Haerbin Electrical Carbon Factory, Harbin 150000, China; m13359498992_1@163.com

**Keywords:** carbon composite, thermal shock, porosity, compressive strength, electrical resistivity

## Abstract

Carbon composite is widely used in various fields, including the aerospace industry, electrical engineering, transportation engineering, etc. For electrified railways, the pantograph strip utilizes carbon composite as the current collector, which might bear multiple impacts from electrical, mechanical, or thermal aspects, from unwanted arcing, rain, and other diverse operation conditions. In this paper, a thermal shock damage experiment on the carbon composite of a pantograph strip was carried out. The thermal shock processes were realized by the adoption of muffle furnace heating and water cooling. The effect of thermal shock processes on carbon strip porosity, compressive strength, electrical resistivity, and surface topography were studied. In order to verify the mechanism of thermal shock damage to the pantograph strip, the porosity of the pantograph strip is discussed in detail. The results showed that the thermal shock process increased the porosity of the carbon strip and caused reductions in compressive strength and electrical resistivity. The multiple thermal shock processes caused irreversible damage to the pantograph strip, which was attributed to the spillover and scouring of large quantities of water vapor in the pores.

## 1. Introduction

Carbon composite has been widely used in various fields, including the aerospace industry [1], electrical engineering [2], transportation engineering [3], etc. Particularly, carbon composite has been chosen as the key component of the current collector in electrified railway pantograph–catenary systems. For example, in a high-speed electrified railway, the bullet train constantly acquires electric energy from the overhead catenary lines through a pantograph strip. As the unique piece of equipment used to acquire current, the pantograph strip is key to guaranteeing that the electric locomotive will run safely and steadily [4,5]. The pantograph strip is designed as a kind of lossy material for the purpose of protecting the catenary line. Usually, loss from the pantograph strip comes from mechanical friction and arc erosion, which results mostly in a service performance reduction and even causes the fracture of the pantograph strip [6,7].

Damages to the carbon composite might occur under repeated hot and cold alternating conditions, which is the case for electric locomotives running in changeable weather and pantograph off-line arcing conditions. On one hand, the mechanical friction, Joule heat, and arc erosion increase the temperature of pantograph strip. On the other hand, rainwater would reduce the temperature drastically on a rainy day. The hot and cold alternating conditions would aggravate the damage to the pantograph strip during the operation of the electric locomotive. This kind of damage is named “thermal shock damage” hereinafter.

Thermal shock damage has been found in architecture materials such as rock and concrete. Research has shown that thermal shock damage can have serious adverse effects on the static performance parameters of granite [8,9]. It was proved that high-strength concrete would begin to crack when suffering from a temperature step frequently changing from 18 to 20 °C [10,11]. It was suggested that the different thermal expansion coefficients of the material components would lead to intergranular compression and tensile force, which might result in a specific microcrack network [12,13,14,15,16,17,18,19,20,21].

As mentioned above, the key parts of the pantograph strip are mostly made up of carbon composite, which usually contains graphite powder, resin adhesive, petroleum coke, etc. [14,15]. Due to the thermal expansion coefficients of these materials being inconsistent, there would be different expansion degrees between the different crystals after the same thermal shock processing, which most likely causes enlargement of the pores and microcracks. At last, a certain degree of damage to the pantograph strip will take place.

In this paper, thermal shock damage experiments on the carbon composite used in pantograph strips were carried out. The thermal shock processes were realized by a muffle furnace heating and water cooling strategy. The effects of thermal shock processes on the carbon composite porosity, compressive strength, electrical resistivity, and surface topography were respectively examined. Results after one cycle of thermal shock impact as well as after multiple cycles of thermal shock impact were compared. A discussion of the mechanism of thermal shock damage to the carbon composite is presented from the microscopic view of pore evolution. In the final part, the conclusions are drawn.

## 2. Materials and Methods

The test block of carbon composite was selected from pantograph strip CY280, which is widely used in high-speed railways in many countries such as China, Japan, and Germany. The size of the test block was 20 mm × 40 mm × 200 mm. The average value of two or three samples was taken as the final test value. The material composition proportions and the thermal expansion coefficients of the pantograph strip are shown in Table 1.

**Figure 1:**
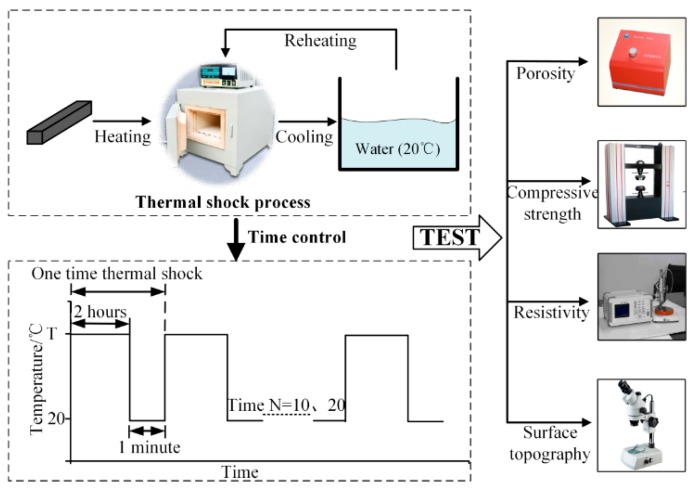
Schematic diagram of the experiment setup.

A schematic diagram of the experimental setup is shown in Figure 1. The thermal shock process was simulated using a muffle furnace and a sink. The muffle furnace (Sx2, GuangHe Co., Zhejiang, China) with a chamber (size 200 × 120 × 80 mm^3^) was used to heat the carbon strip; the maximum heating temperature could be adjusted. The sink was filled with cooling water at 20 °C and was used to cool down the carbon strip. The sink’s diameter was 400 mm and its depth was 800 mm. At present, the overall temperature rise of the pantograph strip during train operation can generally reach about 400 °C [13]; therefore, samples were heated for 2 h at 100, 200, 300, 350, or 400 °C, and were then cooled for 1 min in 20 °C water. After cooling, the carbon strip was reheated using the muffle furnace several times again, to examine the accumulative effect. 

Some parameters such as porosity, compressive strength, electrical resistivity, and surface topography were tested. The porosity was deduced from the true density and bulk density; the true density was measured using a true density meter (JL-1206, JingXin Co., Chengdu, China). The compressive strength was tested using a hydraulic universal testing machine (HF-600E, Kehui Co., Jinan, China) with a loading speed of 1000 N/s. The electrical resistivity of the test block was measured using a four-probe resistivity tester (RTS-8, Jingge Electronic Co., Suzhou, China) which adopted four electrode probes to avoid the nonuniform current and increased the electrical resistivity test accuracy. The surface topography of the test block was observed using optical microscopy (SZM-45T2, Youte Electronic Co., Shenzhen, China) with a maximum magnification of 1600×.

## 3. Results and Discussions

This section is divided into results on the porosity (Section 3.1), the static performance parameters (Section 3.2), and the damage performance (Section 3.3). A discussion of the mechanism of thermal shock damage to the carbon composite is presented based on the systemic experimental results.

### 3.1. Porosity

Porosity is one of the basic physical properties of the pantograph strip. It can be classified into different types such as total porosity, open porosity, and connected porosity, etc. Here, we approximated the law of change in the strip porosity by considering the change in open porosity only [3,22].

In order to measure the open porosity of the test block, the method according to ISO 5017-2013 was used [9]. The porosity ϕ_open_ was defined as

(1) $$\phi_{\mathrm{open}}=\frac{M_{3}-M_{1}}{M_{3}-M_{2}}$$

where *M*_1_ is the test block mass in air, *M*_2_ is the test block mass in water after full water seepage, *M*_3_ is the test block mass in the air after full water seepage.

**Figure 2:**
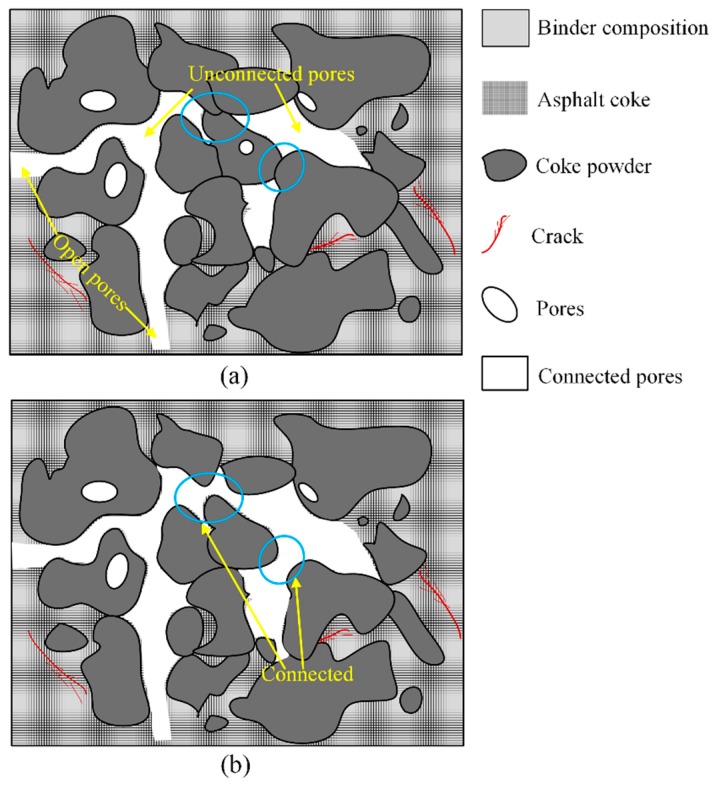
Different types of micropore (**a**) before temperature increase and (**b**) after temperature increase.

The measured results of the Archimedes drainage method when the pantograph strip goes through only one cycle of thermal shock are shown in Table 2. The mass *M*_1_ of the test block in air was 256 g, while the mass *M*_3_ of the test block in air after full water seepage at the thermal shock temperature of 20 °C was 264 g; this showed that the carbon strip was not sealed completely, and there were some fixed open pores in it. With the increase of the thermal shock temperature, the mass *M*_3_ exhibited as a trend of growth. This showed that the water permeability of the carbon strip increased. The pores in the carbon strip were expected to expand gradually after the temperature increase. The sealed pores became open pores and the unconnected pores connected with each other [23,24,25], as Figure 2 shows. As a result, the open porosity factor of the carbon strip increased.

**Figure 3:**
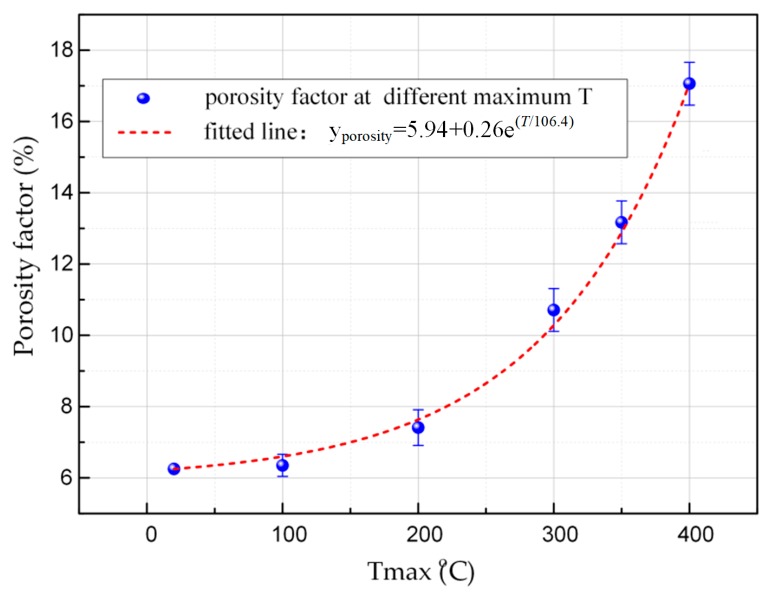
The variation of porosity with thermal shock temperature after a single thermal shock.

The variation of porosity at different maximum thermal shock temperatures is shown in Figure 3. The results showed that porosity increased with the thermal shock temperature increase. The change in the porosity was not remarkable until the thermal shock temperature reached about 200 °C, which indicated that the thermal expansion of pores and the opening of microcracks were not obvious below 200 °C. When the thermal shock temperature reached 300 °C, the porosity began to rise sharply. It increased from 6.25% to 10.91% when the thermal shock temperature reached 400 °C. Because the pantograph carbon strip is a kind of heterogeneous materials, the thermal properties of its internal aggregate and binder are inconsistent, so intercrystalline compression and tensile force could be produced in the state of hot and cold alternate processes; this would make its crystallinity status worse, leading to the expansion of the pores in the pantograph carbon strip.

It should be noted that the porosity recovered to the original state of 6.25% after cooling when the pantograph strip went through just one cycle of thermal shock. The carbon strip materials do not change in chemical properties after one shock process, and the internal thermal stress was still far less than the elastic limit of this kind of material. The expanded pores were able to shrink and recover again after cooling. Elastic deformation occurred in the pantograph strip during this experiment.

### 3.2. The Static Performance Parameters

#### 3.2.1. Compressive Strength

**Figure 4:**
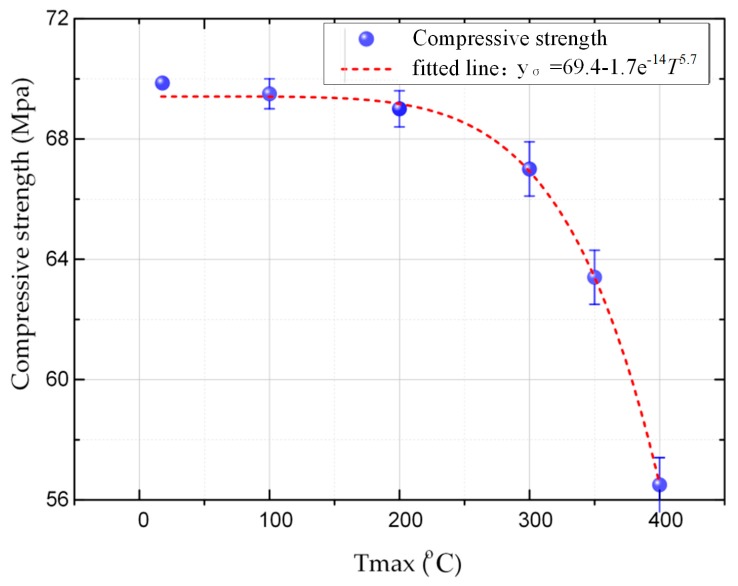
The variation of compressive strength with thermal shock temperature after a single thermal shock.

The variation of compressive strength at different maximum thermal shock temperatures is shown in Figure 4. The compressive strength of the carbon strip decreased with increasing thermal shock temperature. The results showed that the compressive strength was negatively correlated with the thermal shock temperature. Similar to the previous porosity variation, the change in compressive strength was not significant until the maximum thermal shock temperature reached about 200 °C. The compressive strength began to decrease sharply after the thermal shock temperature reached 300 °C. It decreased from 70.05 to 56.58 MPa when the thermal shock temperature reached 400 °C. However, the carbon strip still could recover its original compressive strength of 70.05 MPa gradually when the carbon strip went through only one thermal shock process. The accumulative effects of thermal shock to the carbon strip which resulted in unrecoverable damages are examined later in Section 3.3.

It is easy to see from Figure 3 and Figure 4 that the compressive strength was negatively correlated with the porosity. They both began to change at a temperature of 200 °C. Due to the temperature increase, the pores in the pantograph strip expanded gradually, which made the continuity of this material decrease. When the pantograph strip was subjected to impact loading, the stress could not be dispersed effectively, and the impact resistance decreased gradually [26]. However, the porosity of the pantograph strip was recovered after cooling when it went through only once thermal shock process. Similarly, the compressive strength recovered to its original state after cooling, which showed that the porosity is an important parameter in the mechanical performance of pantograph strips.

#### 3.2.2. Electrical Resistivity

**Figure 5:**
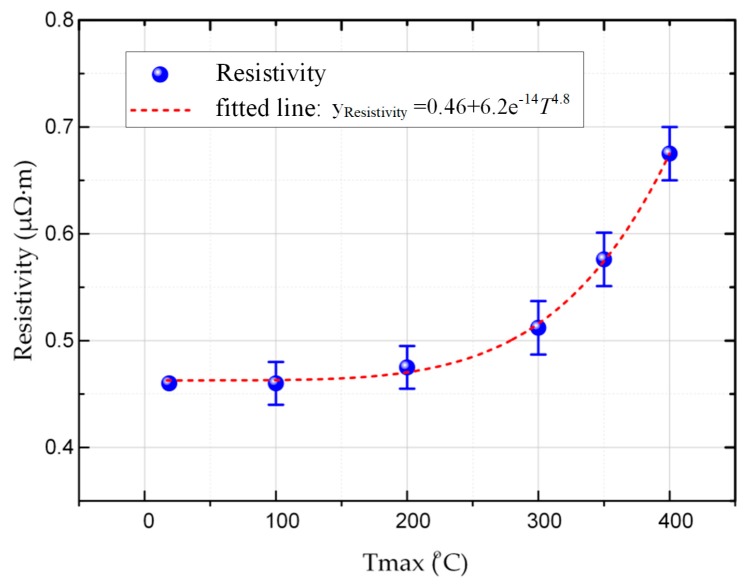
The variation of electrical resistivity with thermal shock temperature after a single thermal shock.

The variation of the electrical resistivity with thermal shock temperature is shown in Figure 5; the electrical resistivity of the carbon strip increased with increasing thermal shock temperature. The change in electrical resistivity was not significant when the thermal shock temperature was below 200 °C. The electrical resistivity began to increase rapidly when the thermal shock temperature reached 300 °C. The electrical resistivity increased from 0.465 to 0.675 μΩ∙m when the thermal shock temperature reached 400 °C. However, it recovered to the original electrical resistivity of 0.465 μΩ·m gradually when the pantograph strip went through only once thermal shock process.

The electrical resistivity was positively correlated to the porosity, as shown in Figure 3 and Figure 5. The density of the electrons and electron holes decreased as the porosity increased, thus lowering the transmitted current [27]. In addition, the pores were also static obstacles for the conduction of electrons: once the electrons meet with the pores, free electrons are scattered by more pores during conduction, thus hindering the movement of conducted electrons [28]. This caused a drop in the electrical conductivity of the pantograph strip. The change in electrical resistivity after the thermal shock was similar to that in compressive strength: it returned to its original state after cooling, which once again indicated that porosity is an important parameter in the electrical performance of pantograph strips.

### 3.3. Damage Performance

**Figure 6:**
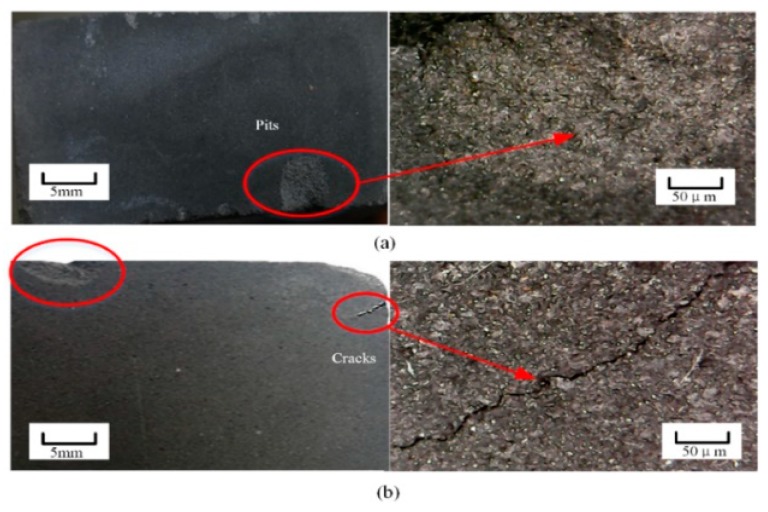
The surface morphology of the pantograph strip after repeated thermal shock.

One cycle of thermal shock had a significant effect on the porosity of the carbon strip for a limited period of time, which resulted in changes in compressive strength and electrical resistivity. In order to verify whether thermal shock could produce irreversible damage to the carbon strip, multiple repeated thermal shock experiments at the temperature of 300 °C were also carried out. As expected, unrecoverable variation of the abovementioned parameters was confirmed. Furthermore, macroscopic surface change was observed. The surface topography of the carbon strip after repeated thermal shock is shown in Figure 6. It can be seen from the figure that the surface of the carbon strip was uneven and appeared to have falling blocks after ten rounds of repeated thermal shock. After twenty rounds of thermal shock, the phenomenon of falling blocks on the edge of the strip was more obvious, and the pits increased gradually with the formation of visible and obvious cracks. This indicated that the damage to the pantograph strip was aggravated by increasing number of thermal shock rounds. Further, the elastic deformation of pore expansion caused by the repeated thermal shock accumulated gradually and eventually transformed into plastic deformation. 

In order to find out the reason for irreversible damage to the carbon, an extra experiment was performed. The samples were heated for 2 h at 300 °C and cooled for 1 min in 20 °C water. Then, the samples were dried for 15 min at 60 °C before the next thermal shock process. The drying process was used to remove the water in the carbon strip smoothly. As a result, the carbon strip could be reheated without the effect of water vapor.

With the added drying process, the porosity of the pantograph strip increased less. The porosity of the carbon strip with the drying process was 19.11% from the measured data, different from the porosity of 22.43% of the carbon strip without the drying process. From the comparison, the water in the pores was considered to be the critical factor for the thermal shock damage. Due to the high temperature, the water boiled and formed vapor very quickly. The great pressure from the vapor broke through the pores, and the spillover and scouring of large quantities of water vapor in the pores led to a rapid increase in the average size of pores and microcracks in the strip. It can be seen that the spillover and scouring of water vapor is an important factor leading to the rapid increase of pores and microcracks in the pantograph strip.

## 4. Conclusions

The thermal shock damage characteristics of a carbon composite were experimentally studied. The thermal shock processes were simulated using a high-temperature muffle furnace and a sink with 20 °C water. Maximum thermal shock temperatures of 100, 200, 300, 350, and 400 °C were applied to the carbon composite. Characteristic parameters such as porosity, compressive strength, and electrical resistivity experienced a sharp change once the maximum thermal shock temperature reached about 200 °C. This change was recovered after the carbon composite was cooled if it went through only one cycle of the thermal shock process. With multiple thermal shock processes, irreversible damage to the carbon composite occurred. Cracks and pits appeared after about 20 rounds of thermal shock processes.

The porosity is considered to be a critical parameter in the process of thermal shock damage and had significant effects on the carbon static performance, such as the compressive strength and electrical resistivity. The water in the pores was evaporated at high temperature, and the spillover and scouring of large quantities of water vapor in the pores caused a rapid increase in the average size of pores and microcracks in the strip.

## Figures and Tables

**Table 1:** Material composition proportions and the thermal expansion coefficients of samples.

Components	Graphite Powder	Resin Adhesive	Petroleum Coke	Mid-Temperature Pitch
Thermal Expansion Coefficient (K^−1^)	5.5 × 10^−^^6^	1 × 10^−5^	2.6 × 10^−6^	5.5 × 10^−4^
Proportion (mass fraction, %)	30–60%	8–10%	8–32%	15–35%

**Table 2:** The measured results of the Archimedes drainage method.

Thermal Shock Temperature (°C)	*M*_1_ (g)	*M*_2_ (g)	*M*_3_ (g)
20	256	104	264
100	256	104	264
200	256	106	268
300	256	109	274
350	256	111	278
400	256	115	285
